# The false smut pathogen *Ustilaginoidea virens* requires rice stamens for false smut ball formation

**DOI:** 10.1111/1462-2920.14881

**Published:** 2019-12-11

**Authors:** Jing Fan, Jie Liu, Zhi‐You Gong, Pei‐Zhou Xu, Xiao‐Hong Hu, Jin‐Long Wu, Guo‐Bang Li, Juan Yang, Yu‐Qiu Wang, Yu‐Feng Zhou, Shuang‐Cheng Li, Li Wang, Xiao‐Qiong Chen, Min He, Ji‐Qun Zhao, Yan Li, Yan‐Yan Huang, Dong‐Wei Hu, Xian‐Jun Wu, Ping Li, Wen‐Ming Wang

**Affiliations:** ^1^ State Key Laboratory of Crop Gene Exploration and Utilization in Southwest China Rice Research Institute, Sichuan Agricultural University Chengdu 611130 China; ^2^ College of Agronomy Sichuan Agricultural University Chengdu 611130 China; ^3^ State Key Laboratory of Rice Biology Biotechnology Institute, Zhejiang University Hangzhou 310058 China; ^4^ Collaborative Innovation Center for Hybrid Rice in Yangtze River Basin Sichuan Agricultural University Chengdu 611130 China

## Abstract

Rice false smut has emerged as a serious grain disease in rice production worldwide. The disease is characterized by the transformation of individual rice florets into false smut balls, which is caused by the fungal pathogen *Ustilaginoidea virens*. To date, little is known about the host factors required for false smut ball formation by *U*. *virens*. In this study, we identified histological determinants for the formation of false smut balls by inoculating *U*. *virens* into rice floral mutants defective with respect to individual floral parts. The results showed that *U*. *virens* could form mature false smut balls in rice floral mutants with defective pistils, but failed to develop false smut balls in the *superwoman* mutant lacking stamens, identifying that *U*. *virens* requires rice stamens to complete its infection cycle. Comparative transcriptome analysis indicated a list of candidate host genes that may facilitate nutrient acquisition by *U*. *virens* from the rice stamens, such as *SWEET11*, *SWEET14* and *SUT5*, and genes involved in the biosynthesis of trehalose and raffinose family sugars. These data pinpoint rice stamens as the key target organ of *U*. *virens* infection and provide a valuable starting point for dissecting the molecular mechanism of false smut ball formation.

## Introduction

Rice false smut disease is caused by the ascomycete fungal phytopathogen *Ustilaginoidea virens* (Cooke) Takahashi (teleomorph: *Villosiclava virens*) (Fan *et al*., 2016a). This disease has been reported with increasing occurrence in most rice production areas worldwide (Brooks *et al*., 2009; Ladhalakshmi *et al*., 2012; Jecmen and TeBeest, 2015). It not only causes considerable losses in terms of grain yield and quality but also acts as a health hazard to humans and animals due to poisonous mycotoxins contaminating the rice grains and straws of infected plants. The mycotoxins, including ustiloxins and ustilaginoidins, are produced from the fungal colonies in rice florets, called false smut balls (Koiso *et al*., 1994; Nakamura *et al*., 1994; Zhou *et al*., 2012; Lu *et al*., 2015; Wang *et al*., 2016a; Sun *et al*., 2017; Wang *et al*., 2017; Wang *et al*., 2019).

The false smut ball is the only visible symptom of rice false smut disease (Fan *et al*., 2016a). The process of false smut ball formation has been extensively investigated and can be summarized as follows. At the late booting stage of rice, spores of *U*. *virens* germinate on the surface of a rice spikelet (Ashizawa *et al*., 2012). Then, hyphae of *U*. *virens* extend into the inner space of the spikelet though the gap between the lemma and the palea and infect inner floral parts, including stamen filaments, lodicules and pistil (Ashizawa *et al*., 2012; Tang *et al*., 2013; Li *et al*., 2013b; Hu *et al*., 2014; Song *et al*., 2016). During infection, *U*. *virens* interrupts the process of rice flowering and fertilization and hijacks host nutrients for mycelial growth and false smut ball formation (Fan *et al*., 2015; Song *et al*., 2016). False smut balls are covered with chlamydospores that become orange or green (Wang *et al*., 2019) and generate sclerotia when exposed to large day/night temperature differences (Fan *et al*., 2016b). As chlamydospores and sclerotia are produced from false smut balls and are the primary inocula of the rice false smut disease (Ikegami, 1960; Fan *et al*., 2010; Yong *et al*., 2018), understanding the factors involved in the formation of false smut ball is significant for controlling the disease.

To dissect the mechanism of false smut ball formation, the first step is to identify the infection sites of *U*. *virens* in rice flowers. It has been reported that *U*. *virens* primarily infects stamen filaments and, to a lesser extent, the lodicules (Tang *et al*., 2013). *U. virens* has also been reported to infect stigmas and styles (Song *et al*., 2016). Occasionally, *U*. *virens* hyphae can infect the ovary (Li *et al*., 2013b; Song *et al*., 2016). Several studies have consistently found that at all the observed infection sites *U*. *virens* extends only intercellularly and cannot form infection structures such as appressoria and haustoria (Tang *et al*., 2013; Hu *et al*., 2014; Song *et al*., 2016). So far, *U*. *virens* infection sites have not been detected in other floral parts such as anthers, lemma, palea or rachilla (Tang *et al*., 2013; Song *et al*., 2016). Therefore, we speculated that successful colonization of *U*. *virens* may require the presence of stamen filaments, lodicules or pistil. Nevertheless, it is still an open question as to which floral parts are essential to support false smut ball formation by *U*. *virens*.

In the current study, we investigated the events in detail at early infection stages by utilizing an efficient artificial inoculation system with a Green Fluorescent Protein (GFP)‐tagged *U*. *virens* isolate and a highly susceptible rice accession. Based on the statistical analysis of data obtained by examining thousands of spikelets, we found that *U*. *virens* preferentially attacked rice stamens. Moreover, we performed artificial inoculation on several floral rice mutants deficient with respect to either stamens or pistils and demonstrated that rice stamens were essential for the formation of false smut ball. To identify potential molecular determinants involved in false smut ball formation, we further conducted RNA‐Seq analysis on rice stamens infected with *U*. *virens*, which provided novel insights into the underlying mechanisms of nutrient acquisition from rice florets by *U*. *virens*.

## Results

### *Ustilaginoidea virens preferentially attacks stamen filaments of rice*

**Figure 1:**
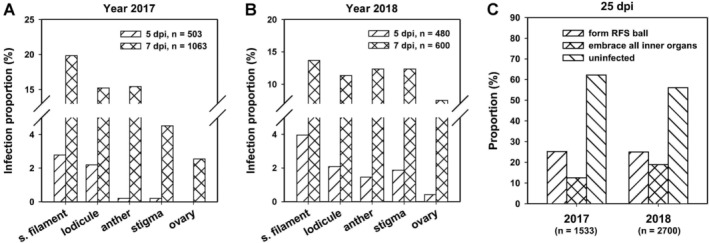
The proportion of rice spikelets with *Ustilaginoidea virens* infection in each floral part across the infection process. In the year 2017 (A) and 2018 (B), a GFP‐tagged *U*. *virens* isolate P4 was inoculated into panicles of rice accession Pu6 at late booting stages. Spikelets were collected from inoculated panicles at indicated time points and examined under a fluorescence microscope. The infection status of P4 in each floral part of each spikelet was recorded. The infection proportion was calculated for each floral part. C. At 25‐day post‐inoculation (dpi), thousands of spikelets were sampled from over 15 inoculated panicles for counting the number of rice false smut balls or checking the infection status of P4 in spikelets without forming false smut balls. s. filament, stamen filament; embrace all inner organs, P4 mycelia embrace all inner floral parts including stamen filaments, anthers, lodicules, stigmas and ovary, but without false smut ball formation.

**Figure 2:**
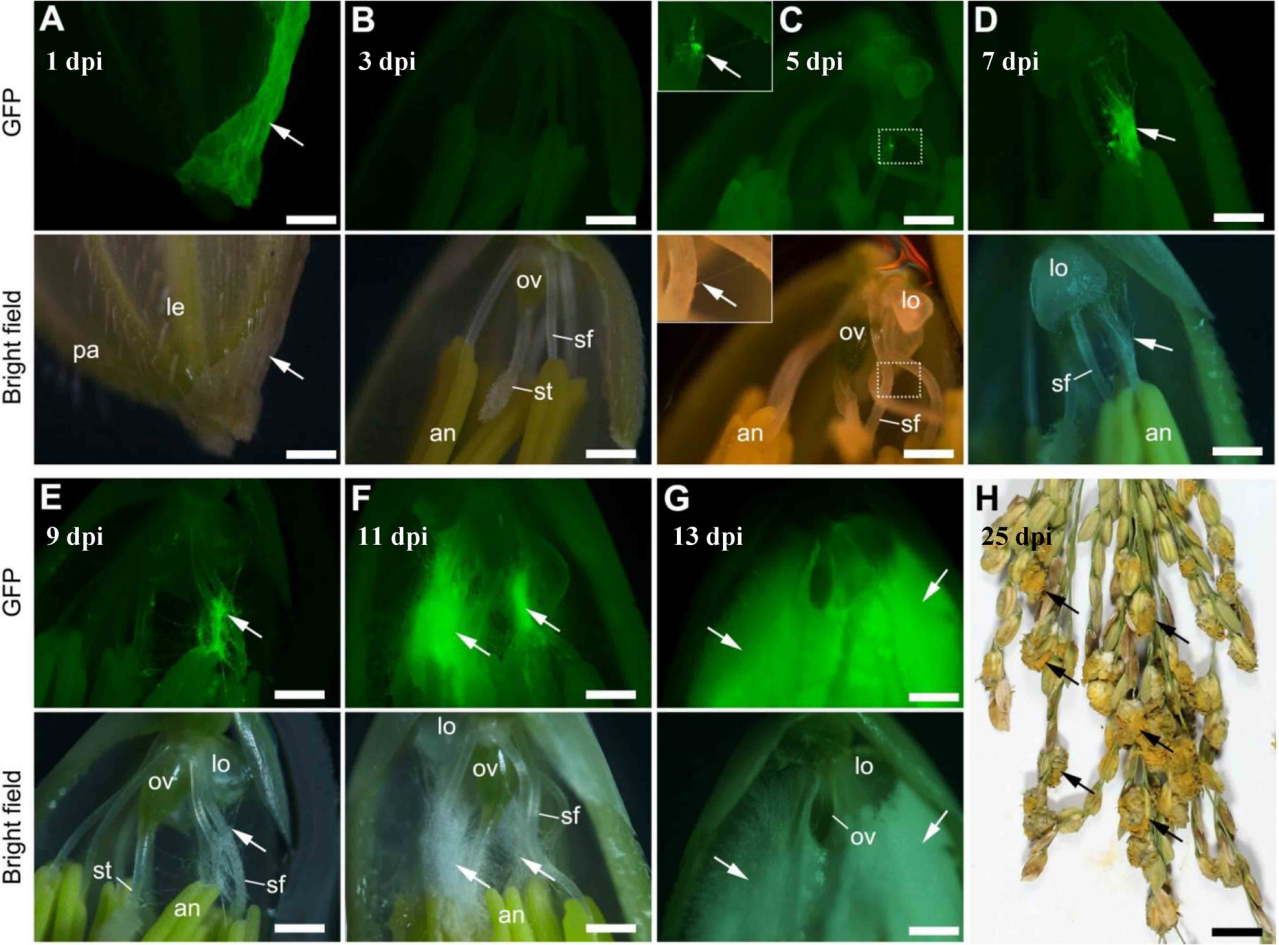
The remodelled infection process of *Ustilaginoidea virens* in rice florets based on statistical data. A GFP‐tagged *U*. *virens* isolate P4 was inoculated into panicles of rice accession Pu6 at the late booting stage. Spikelets were collected from inoculated panicles at indicated time points and examined under a fluorescence microscope. Based on the infection proportion data as shown in Fig. 1, representative images of P4 infection in rice floret at each time point were selected to construct a stepwise infection process. P4 hyphae were only detected on the surface of spikelet at 1 (A) and 3‐day post‐inoculation (dpi) (B). At 5 dpi, the pathogen hyphae extended into the inner space of spikelet and first invaded stamen filaments (C). The hyphae grew towards lodicules and anthers at 7 dpi (D) and reached the stigmas and ovary at 9 dpi (E). Subsequently, the pathogen continued to grow and embrace all the inner floral parts from 11 (F) to 13 dpi (G). At 25 dpi, numerous mature false smut balls were formed in each infected panicle (H). sf, stamen filament; lo, lodicule; an, anther; st, stigma; ov, ovary; le, lemma; pa, palea. Insets in (C) represent an enlargement of image areas marked with dotted line rectangles. White and black arrows indicate hyphae and false smut balls respectively. Bar size = 500 μm (A–G), 1 cm (H).

To identify the key target site of *U*. *virens* in rice spikelets, we first examined whether *U*. *virens* showed any preference for specific floral parts during infection. We monitored the infection process at early infection stages in detail by utilizing a GFP‐tagged *U*. *virens* strain P4 and a highly susceptible rice accession Pujiang6 (Pu6) for artificial inoculation. We examined the outer surface and inner space of more than 500 rice spikelets collected from multiple inoculated panicles every 2 days post‐inoculation (dpi) and recorded the infection status of *U*. *virens* in each floral part of each spikelet. Our data demonstrated that P4 hyphae were restricted to the outer surface of all the examined spikelets at 1 and 3 dpi, extending to the inner floral parts at 5 dpi (Figs 1 and 2A–C).

Taking the data from year 2017 as an example (Fig. 1A), P4 hyphae reached the stamen filaments of 2.78% of the examined spikelets and the lodicules in 2.19% of the examined spikelets at 5 dpi. The value fell to 0.2% for both anthers and stigmas at 5 dpi. At 7 dpi, the percentage infection increased rapidly, with P4 hyphae reaching all the inner floral parts (Fig. 2D), at percentages of 19.85, 15.24, 15.43, 4.52 and 2.54 for stamen filaments, lodicules, anthers, stigmas and ovaries respectively (Fig. 1A). The percentage infection was the least for ovaries at both 5 and 7 dpi (Fig. 1A). At 9 dpi, P4 hyphae had reached all inner floral parts of most (over 86%) of the infected spikelets (Fig. 2E). Subsequently, fungal growth increased rapidly to embrace all the inner floral parts (Fig. 2F and G). Although the values of infection proportions varied between years 2017 and 2018, similar trends were observed, i.e. the proportion was the highest for P4 hyphae attacking stamen filaments, followed by lodicules, with ovaries showing the least pathogen colonization (Fig. 1B). These data indicate that *U*. *virens* preferentially attacked stamen filaments.

We also monitored the infection status of P4 in rice florets of Pu6 at a very late infection stage (25 dpi), by which time mature false smut balls had already formed (Fig. 2H). We found that approximately 25% of inoculated spikelets had been converted into false smut balls in both 2017 and 2018 (Fig. 1C). Interestingly, 12.5%–18.9% of the examined spikelets had P4 mycelia embracing all the inner floral parts but were not converted into false smut balls (Fig. 1C). These observations indicate that not all infected spikelets could develop into false smut balls, but colonization of all the inner floral parts is the prerequisite for the conversion of a spikelet into a false smut ball. Therefore, it is important to determine which floral part needs to be colonized in order to achieve the formation of false smut balls.

### *Ustilaginoidea virens fails to develop false smut balls in stamen‐/pistil‐deficient rice mutant*

**Figure 3:**
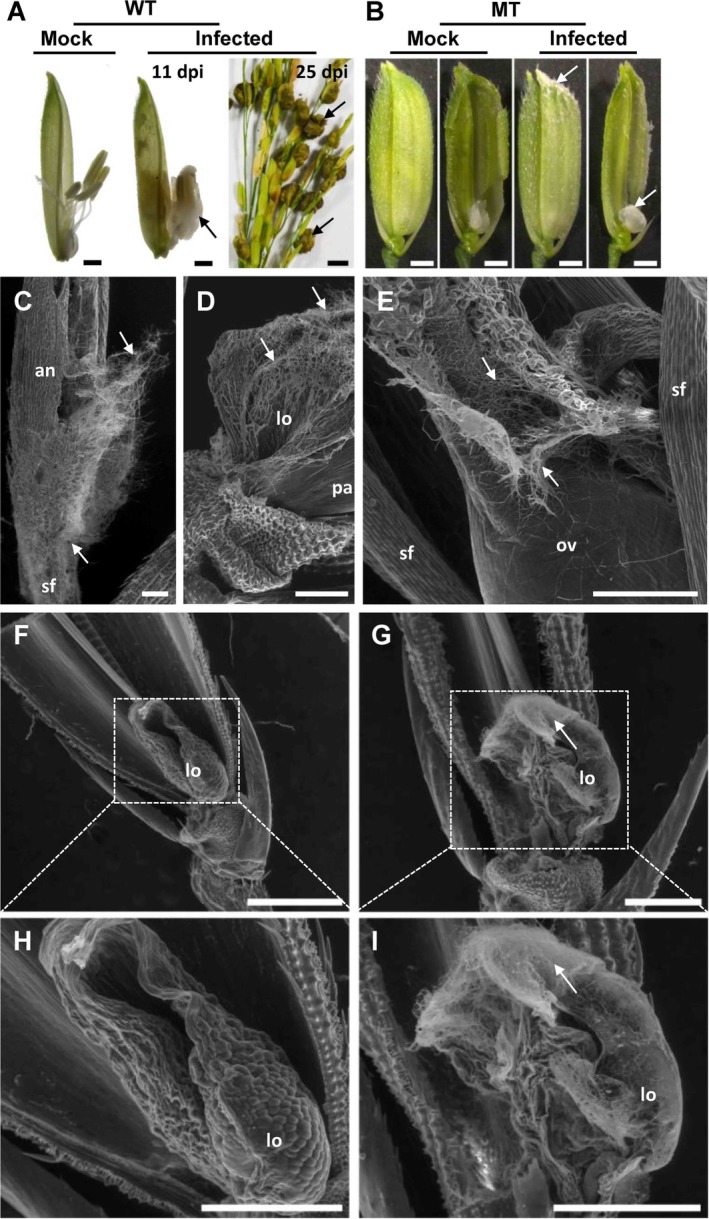
Infection of *Ustilaginoidea virens* in a stamen‐/pistil‐deficient rice mutant. A. *U.virens* isolate PJ52‐2‐5 infected an *indica* rice accession Yixiang1B (wild‐type, WT) and formed false smut balls (black arrows). B. A stamen‐/pistil‐deficient rice mutant (MT) was derived from an EMS mutagenesis mutant library of Yixiang1B. In the inner space of MT spikelet, only lodicules were present. PJ52‐2‐5 failed to form false smut balls in MT but did grow on the lodicules with visible fungal mass (white arrows). C–E. Scanning electron microscopy (SEM) analysis of Yixiang1B floret infected with *U*. *virens* at 11 dpi (day post‐inoculation). F, H. SEM image of MT floret without *U*. *virens* infection. G, I. SEM image of MT floret infected with *U*. *virens* at 11 dpi. an, anther; lo, lodicule; ov, ovary; pa, palea; sf, stamen filament. White arrows indicate *U*. *virens* hyphae. Bar size = 1 cm (right panel in A), 1 mm (A, B), 200 μm (C–E) and 500 μm (F–I).

We screened floral organ deficient mutants from our previous ethyl methanesulfonate (EMS)‐mutagenesis library derived from an *indica* rice Yixiang1B (Liao *et al*., 2018), for their ability to form false smut balls following inoculation with *U*. *virens* PJ52‐2‐5 (Fig. 3A). The mean number of false smut ball per diseased panicle reached 22 and the disease incidence was up to 85% (Table 1), following inoculation of PJ52‐2‐5 onto wild‐type Yixiang1B, indicating compatible interaction.

**Table 1:** Disease assay data of rice floral mutants inoculated with *U*. *virens*.

Material	Background	Number of inoculated panicles	Number of diseased panicles^a^	Disease incidence (%)	Average number of false smut balls per diseased panicle^b^	Diameter of false smut ball (cm)^b^
Yixiang1B	N/A	27	23	85.19	22.00 ± 0.65	0.84 ± 0.16
Stamen‐/pistil‐deficient mutant	Yixiang1B	30	0	0	N/A	N/A
Stamen‐deficient/multi‐ovary mutant	Yixiang1B	50	0	0	N/A	N/A
W2555	N/A	20	13	65.00	16.38 ± 6.90	0.64 ± 0.09
Pistil‐defective mutant	W2555	20	12	60.00	13.58 ± 5.81	0.92 ± 0.23***
X3107	N/A	17	10	58.82	31.40 ± 18.05	0.53 ± 0.09
*ptb1*	X3107	18	11	61.11	47.36 ± 14.08	0.50 ± 0.03

N/A, not applicable.

a Diseased panicles indicate panicles with false smut balls.

b Data points refer to the mean ± standard deviation. Student's *t*‐test was performed to examine the significance of differences between wild‐type and mutant. ***P‐value < 0.001.

We isolated a floral mutant without any stamens or pistil in its floret but possessing two lodicules (Fig. 3B). We inoculated 30 panicles of this stamen‐/pistil‐deficient mutant with PJ52‐2‐5, but did not observe false smut balls formed from the mutant spikelets, although there was limited pathogen growth on the lodicules of 16 infected spikelets from seven diseased panicles (Fig. 3B; Table 1). Using an environmental scanning electron microscope, we observed hyphae of the pathogen on anthers, stamen filaments, lodicules and ovaries of Yixiang1B (Fig. 3C–E). We also observed hyphae of the pathogen covering the lodicules of the mutant florets (Fig. 3G and I), whereas no hyphae were seen on the mock‐inoculated florets (Fig. 3F and H). These data demonstrate that *U*. *virens* hyphae could still invade into the inner space of the stamen‐/pistil‐deficient mutant spikelets but could not form false smut balls. These results indicate that lodicules could support the limited growth of pathogen hyphae but were not sufficient to support false smut ball development.

### *Ustilaginoidea virens could successfully colonize pistil‐defective rice mutants*

**Figure 4:**
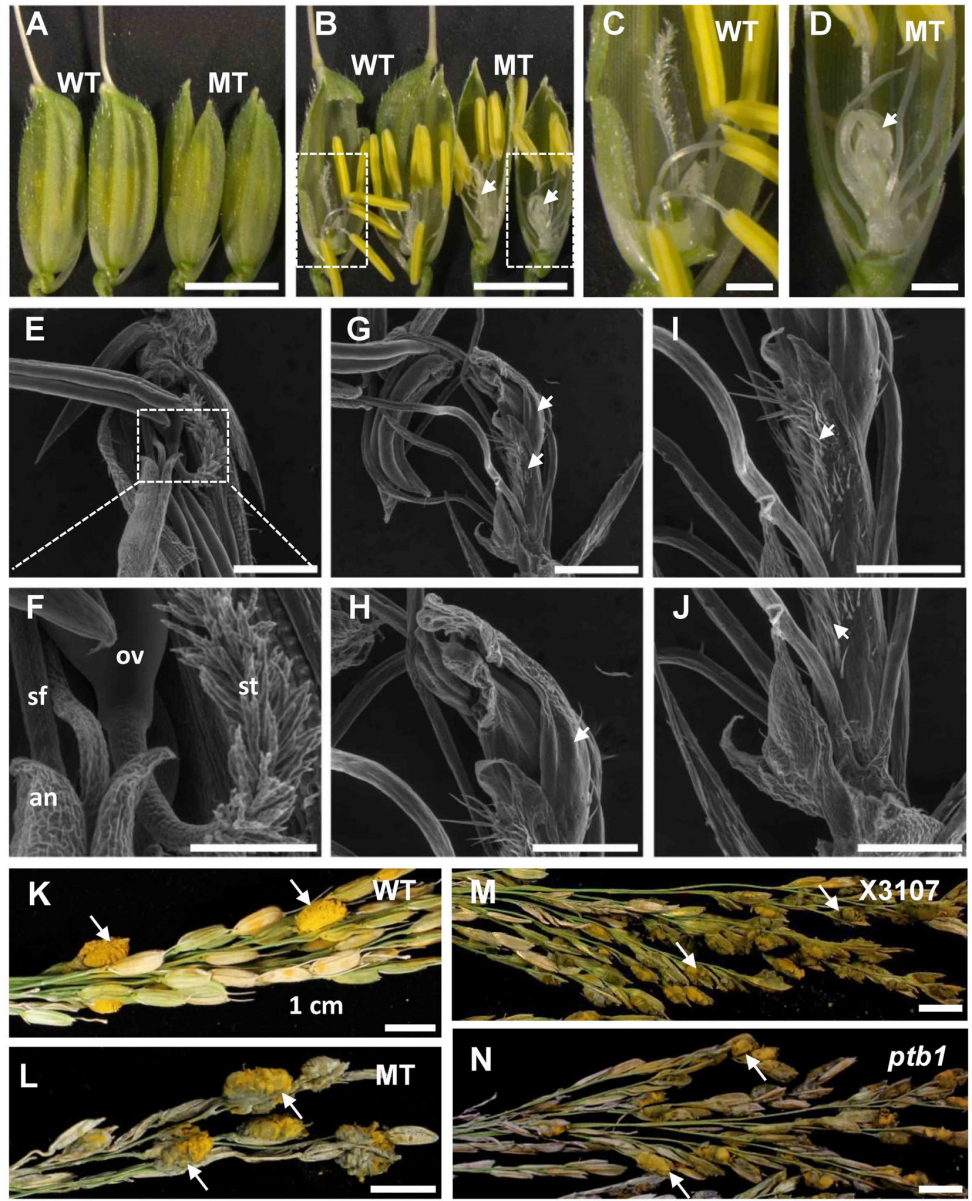
Infection of *Ustilaginoidea virens* in pistil‐defective rice mutants. A, B. Spikelet phenotype of a pistil‐defective mutant derived from accession W2555. Compared with the wild‐type (WT), the mutant (MT) spikelet has normal stamens but no pistils. In addition, the MT spikelet has abnormal lemma and palea, resulting in imperfect closure. Image areas marked with dotted line rectangles were enlarged and presented in (C) and (D). White arrowheads indicate unknown lamellar structures replacing pistil. E–J. Scanning electron microscopy analysis of WT (E, F) and MT (G–J) floral organ. Note that stigmas and ovary were not observed in MT; instead, unknown lamellar structures were present (white arrowheads). K, L. *U.virens* isolate PJ52‐2‐5 was inoculated into panicles of WT (K) and MT (L). Disease phenotype was recorded at 4–5 weeks post‐inoculation (wpi). Mature false smut balls were observed in both WT and MT panicles. M, N. PJ52‐2‐5 was inoculated into panicles of an *indica* rice accession X3107 (M) and a female‐sterile mutant *ptb1* derived from X3107 (N) (Li *et al*., 2013a). Disease phenotype was recorded at 4–5 wpi. Mature false smut balls were observed in both X3107 and *ptb1* panicles. sf, stamen filament; lo, lodicule; an, anther; st, stigma; ov, ovary. White arrows indicate rice false smut balls. Bar size = 5 mm (A, B), 1 mm (C, D, E, G), 0.4 mm (F), 0.5 mm (H–J) and 1 cm (K–N).

To identify the role of rice pistil in *U*. *virens* infection, we inoculated a pistil‐defective mutant, which was a spontaneous mutant derived from rice accession W2555 in our germplasm collection. This floral mutant also exhibited abnormal lemma and palea, resulting in imperfect closure of the spikelet (Fig. 4A). In the innermost whorl of the mutant flower, the pistil was replaced by unknown translucent structures (Fig. 4B). Environmental scanning electron microscopy (ESEM) analysis showed that lamellar structures were generated in the carpel whorl (Fig. 4E–H), in contrast to the normal appearance of W2555 pistil (Fig. 4C and D). In addition, trichome‐like tissues were observed on the lamellar structures of the mutant floret (Fig. 4G and H). As expected, abnormal development of the mutant pistil resulted in sterility of the mutant floret. When this pistil‐defective mutant was artificially inoculated with *U*. *virens* PJ52‐2‐5, mature false smut balls were normally formed in the inoculated panicles, with the ball size being larger than that in the wild‐type W2555 (Fig. 4I; Table 1). These results indicate that *U*. *virens* does not require the presence of a rice pistil for the development of false smut ball.

We also inoculated a pistil‐sterile rice mutant *ptb1* in the rice false smut disease assay. *ptb1* was a spontaneous mutant derived from an *indica* rice X3107 and caused by loss‐of‐function of the *POLLEN TUBE BLOCKED 1* gene (Li *et al*., 2013a). *ptb1* did not allow pollen tube growth towards ovary due to intense callose deposition on the ovule surface, thus resulting in female‐specific sterility (Li *et al*., 2013a). Following inoculation with *U*. *virens* PJ52‐2‐5, *ptb1* supported the formation of false smut balls (Fig. 4J). The average number of false smut ball per diseased panicle and the mean false smut ball size in *ptb1* were not significantly different from those in the wild‐type X3107 (Fig. 4J; Table 1). Taken together, the dysfunction of rice pistil fertilization did not stop *U*. *virens* from developing false smut balls.

### *Ustilaginoidea virens fails to generate false smut balls in stamen‐deficient rice mutant*

**Figure 5:**
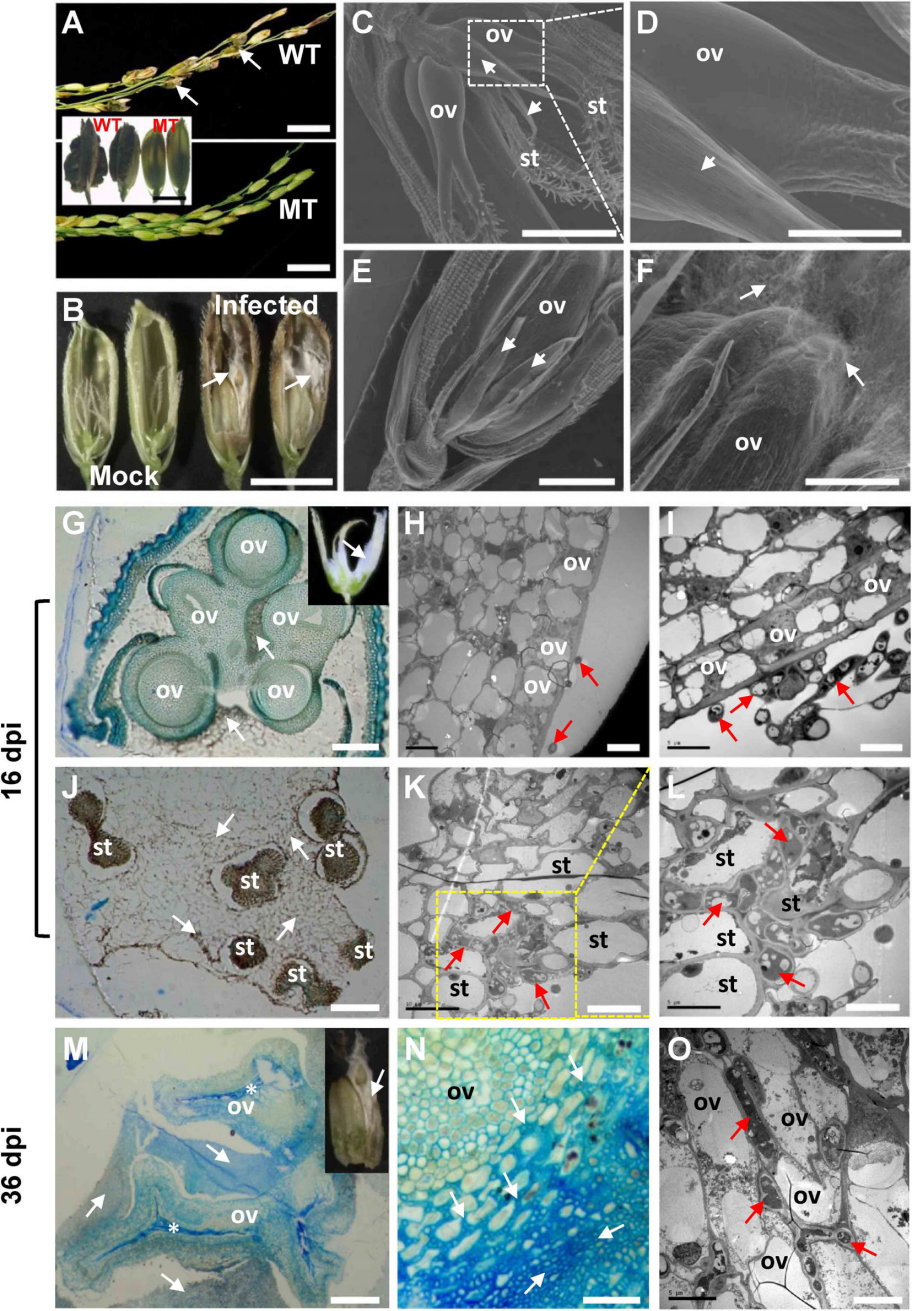
Infection of *Ustilaginoidea virens* in *superwoman* rice mutant. A. Disease phenotype of Yixiang1B (wild‐type, WT) and a *superwoman* mutant (MT) derived from an EMS mutagenesis mutant library of Yixiang1B, at 1 month post‐inoculation of *U*. *virens* isolate PJ52‐2‐5. Mature false smut balls (white arrows) were observed in WT panicles but never observed in MT panicles. B. Observation of inner floral parts of MT spikelets under infected or uninfected conditions. White mycelium was seen (white arrows) and ovaries were enlarged in infected MT spikelets at 36‐day post‐inoculation (dpi). C‐F. Scanning electron microscopy analysis of MT floral organs under uninfected (C, D) or infected (E, F) conditions. Image areas marked with dotted line rectangles in (C) were enlarged and presented in (D). (E) and (F) are views of different parts from the same infected mutant spikelet. Considerable fungal growth (white arrows) was detected on enlarged ovaries in infected spikelets. G–L. Anatomical analysis of infected MT spikelets at 16 dpi, when the ovaries were not enlarged (inset in G). Under light microscopy (G), five ovaries were seen to be surrounded by pathogen hyphae (white arrows). Under transmission electron microscope (H, I), intact ovary cells were observed and were not infected by pathogen hyphae (red arrows). Infection sites on stigmas were examined under both light microscopy and transmission electron microscopy. Large‐scale fungal hyphal accumulation (white or red arrows) was detected around stigmas (J) and in the extracellular space among stigma cells (K, L). M–O. Anatomical analysis of infected MT spikelets at 36 dpi. Note that the infected ovaries were enlarged (inset in M). The pathogen hyphae (white or red arrows) were detected around ovaries (M) and in the extracellular space among cells (N, O). st, stigma; ov, ovary. Arrowheads in Fig. 5C–E indicate palea‐like organs. Arrows indicate *U*. *virens* pathogen. *, collapsed cells. Bar size = 2 cm (A), 5 mm (inset in A and B), 1 mm (C, E), 300 μm (D), 500 μm (F), 5 μm (H, I, L, O), 10 μm (K), 20 μm (N), 200 μm (G, J) and 300 μm (M).

In the EMS‐mutagenesis library of rice accession Yixiang1B (Liao *et al*., 2018), we found another floral mutant without any stamens but with multiple pistils in its floret (Fig. 5), resembling the *superwoman* mutant phenotype (Nagasawa *et al*., 2003). Cytological analysis displayed that the mutant floret had five pistils with partially fused ovaries and two palea‐like organs instead of lodicules (Fig. 5C, D, and G). No stamens or other floral parts were observed. Artificial inoculation analysis showed that this *superwoman* mutant could not support false smut ball formation in spikelets, while the wild‐type Yixiang1B produced mature false smut balls after *U*. *virens* infection (Fig. 5A; Table 1). Interestingly, considerable growth of mycelium was observed on the *superwoman* ovaries, which were enlarged in 19 of 30 infected spikelets at 36 dpi (Fig. 5B, E, and F).

To identify the infection sites of *U*. *virens* in the *superwoman* mutant, inoculated spikelets were collected at 16 dpi (ovaries were not enlarged; inset in Fig. 5G) and 36 dpi (ovaries were enlarged; inset in Fig. 5M), and subjected to anatomical analysis. At 16 dpi, large quantities of *U*. *virens* hyphae were observed surrounding the ovaries (Fig. 5G) and stigmas (Fig. 5J) under a light microscope. Further transmission electron microscopy (TEM) analysis showed that *U*. *virens* hyphae were unable to infect ovary (Fig. 5H and I) but invaded into stigma and extended intercellularly among stigma cells (Fig. 5K and L). Note that the epidermal cells of the ovary were tightly organized, while the stigma cells were irregularly and loosely arranged. At 36 dpi, some ovaries were enlarged in the infected floret, and the remaining ones became degenerated (Fig. 5M). In the middle of the enlarged ovaries, a batch of cells had collapsed. Surprisingly, the outer layer of the enlarged ovaries was found to be infected intercellularly by *U*. *virens* hyphae (Fig. 5N). The intercellular infection of *U*. *virens* in the ovary was further confirmed by TEM analysis (Fig. 5O).

Taken together, these data indicate that *U*. *virens* was unable to form false smut balls in rice mutant florets lacking stamens, despite being able to initiate infection in stigmas or in enlarged ovaries.

### *Ustilaginoidea virens infection results in global transcriptional changes in rice stamens*

As *U*. *virens* needs rice stamens for successful colonization and false smut ball formation (Fig. 1; Fig. 5), we decided to screen for molecular determinants in rice stamens required for *U*. *virens* infection through transcriptome analysis. We collected spikelets from mock‐inoculated and P4‐inoculated panicles of rice accession Pu6 at 9 dpi, because at this infection stage the pathogen hyphae mostly invaded stamen tissues and *U*. *virens*‐infected stamens could be clearly separated from other tissues (Fig. 2E), representing a suitable time point for investigating *U*. *virens*–rice stamen interaction. Stamens were then carefully separated and subjected to RNA‐Seq. In overview, RNA‐Seq generated 41.5–54.7 million clean reads for each replicated sample. Data analysis revealed 4431 differentially expressed genes (DEGs) in infected stamens, compared with control stamens (criteria: *P* < 0.05 and absolute Log_2_ Fold change ≥1). Among the DEGs, 2562 genes were upregulated and 1869 genes were downregulated (Table S1). The data from the DEGs were further subjected to MapMan software for pathway analysis. The results showed that a number of pathways were significantly modulated by *U*. *virens* infection, such as biotic stress, regulation of transcription, receptor kinases, hormone metabolism, secondary metabolism, major carbohydrate metabolism, lipid metabolism, and so forth. (Table S2). For example, DEGs were mostly enriched in pathways of biotic stress (*P* = 1.53E‐11) and receptor kinase signalling (*P* = 2.99E‐11). In particular, 78 out of 93 (83.87%) biotic stress‐related DEGs and 97 out of 117 (82.91%) DEGs encoding receptor kinases were coordinately upregulated in *U*. *virens*‐infected stamens (Table S3). These data indicate that defence responses are transcriptionally activated in rice stamens in response to *U*. *virens* infection; nevertheless, these defence responses could not restrict the pathogen growth in the host tissue.

**Figure 6:**
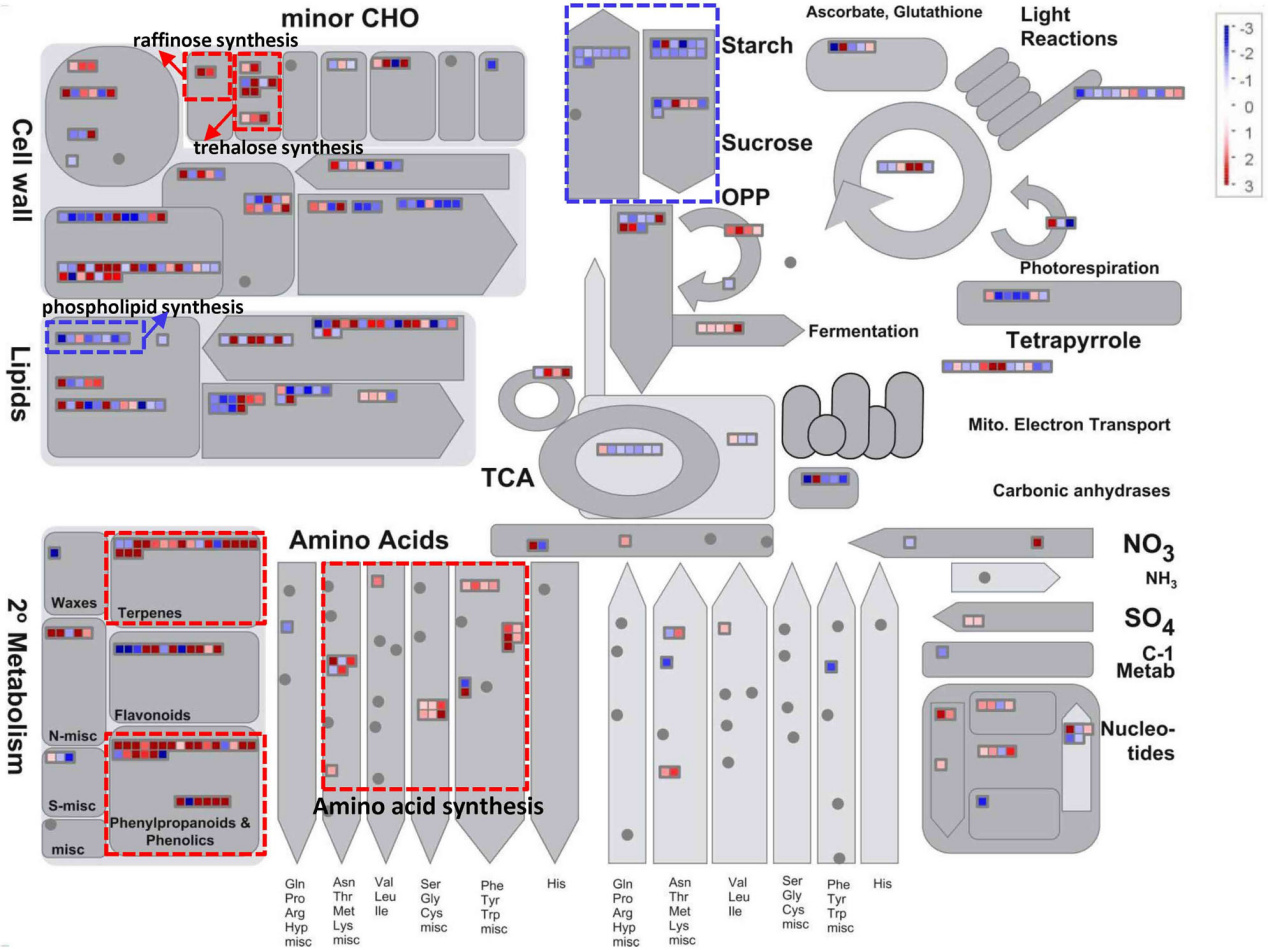
Expression changes of metabolism genes in rice stamens in response to *Ustilaginoidea virens* infection. Rice stamens were collected from mock‐ and *U*. *virens* P4‐inoculated spikelets of rice accession Pu6 and subjected to comparative RNA‐Seq analysis. Differentially expressed genes (DEGs) (absolute Log2 Fold change ≥1 and *P* value < 0.05) were mapped to the Metabolism pathway with MapMan software. Red points represent upregulated genes, while blue points represent downregulated ones. Dotted line rectangles represent pathways of particular interest. Expression data are included in Table S4.

To facilitate visualization of the expression changes of DEGs involved in different metabolisms, we mapped the data of DEGs to metabolism pathways in MapMan. As displayed in Fig. 6, genes involved in amino acid synthesis, phenylpropanoids synthesis and minor carbohydrate metabolism were coordinately upregulated in rice stamens infected with *U*. *virens*, whereas genes involved in starch‐sucrose metabolism and phospholipid synthesis were coordinately downregulated (Fig. 6; Table S4). Particularly, a number of genes responsible for starch synthesis were significantly downregulated, such as *ADP GLUCOSE PYROPHOSPHORYLASE 1* (Os08g0345800), *ADPGLC‐PPASE LARGE SUBUNIT 2* (Os01g0633100), *Granule‐bound starch synthase 1b* (Os07g0412100) and *Soluble starch synthase 3* (Os05g0533600), with expression levels reduced by more than twofold (Fig. 6, Fig. S1; Table S4). On the contrary, genes responsible for the synthesis of raffinose family sugars and trehalose tended to be upregulated. For instance, the expressions of *galactinol synthase 2* (Os07g0687900) and putative *raffinose synthase* (Os06g0172800) were increased by 22.2‐fold and 4.6‐fold (Table S5) respectively. The expressions of nine trehalose synthesis‐related genes were increased by 2.2‐fold–477.7‐fold (Table S4). Moreover, a sucrose export gene *SUT5* (Os02g0576600) was highly upregulated by more than fivefold (Fig. S1; Table S4). Bidirectional sugar transporter genes *SWEET11* (Os08g0535200) and *SWEET14* (Os11g0508600) were also upregulated by 3.4‐fold and 5.8‐fold respectively (Table S1). These data indicate that *U*. *virens* infection may cause inhibition of polysaccharide synthesis but activation of disaccharide/oligosaccharide synthesis in rice stamens, with the sugars that accumulated in the stamen cells being exported into apoplast space to facilitate *U*. *virens* growth.

To validate the RNA‐Seq data, we selected 11 DEGs involved in different pathways for quantitative polymerase chain reaction (qPCR) analysis. The results showed that all the tested genes demonstrated the same trend of expression changes in RNA‐Seq and qPCR experiments (Fig. S2), indicating that the RNA‐Seq data were reliable in this study.

## Discussion

False smut ball is critical for the disease cycle as it generates chlamydospores and sclerotia, representing the primary inocula for this pathogen, but it is detrimental to humans and animals as it produces mycotoxins (Yong *et al*., 2018; Wang *et al*., 2019). Failure of false smut ball formation is believed to interrupt the disease cycle as a result of the depletion of the primary inoculum. Therefore, understanding the mechanism of false smut ball formation is of great potential for controlling the disease. Here, we demonstrated that *U*. *virens* preferentially attacked rice stamen (Fig. 1) and was unable to form false smut balls in rice mutant florets lacking stamens (Fig. 5). In addition, by conducting comparative transcriptome analysis on rice stamens infected with *U*. *virens*, we found that *U*. *virens* modulated host sugar‐ and amino acid‐related metabolic pathways to promote nutrient acquisition.

*U. virens* is a biotrophic fungal pathogen. In an intercellular manner, it infects a subset of rice tissues, primarily stamen filaments, stigmas, the outer layer of lodicules, and epidermal cells of seedling roots and coleoptiles (Ikegami, 1963; Tang *et al*., 2013; Song *et al*., 2016; Prakobsub and Ashizawa, 2017). However, *U*. *virens* could not infect leaves, stems and pedicels of rice (Tang *et al*., 2013; Fan *et al*., 2014). The organ‐specificity of *U*. *virens* infection is suggested to be associated with the ultrastructure and components of cell walls in different rice organs (Yong *et al*., 2016; Rong *et al*., 2017). For example, the packing of cells in stamen filaments is loose and the cell walls are flexible, whereas, in stem and ovary, the cell walls are compact (Tang *et al*., 2013; Yong *et al*., 2016; Rong *et al*., 2017). Therefore, *U*. *virens* may find it easier to colonize stamen filaments, an observation that is supported by our data in this study (Fig. 1). *U. virens* can also easily attack lodicules and stigmas, as demonstrated by the observation that *U*. *virens* hyphae could invade the inner space of stamen‐/pistil‐deficient mutant florets and proliferate on lodicules (Fig. 3), and that a large amount of *U*. *virens* mycelia could be accommodated inside *superwoman* mutant flowers, when only stigmas were infected (inset in Fig. 5G). On the contrary, *U*. *virens* could not infect ovaries with compact cell walls in the *superwoman* mutant (Fig. 5G–I), although *U*. *virens* infection occurred in some ovaries that were enlarged by unknown mechanisms (Fig. 5M–O). These data indicate that *U*. *virens* can infect and proliferate in a few rice organs or tissues, whereas false smut ball formation is restricted to florets.

Which floral part is required for *U*. *virens* to form false smut balls? Previously, Song *et al*. (2016) had proposed that *U*. *virens* infects rice stigmas and styles to mimic ovary fertilization and to continually acquire nutrients for the development of false smut ball, indicating that pistil may be required for false smut ball formation. However, here we found that pistil‐defective rice flowers could still support the false smut ball development (Fig. 4), while stamen‐deficient mutant flowers failed to develop false smut balls after *U*. *virens* infection (Fig. 5). These observations suggest that rice stamen is essential for the formation of false smut balls. How does rice stamen facilitate *U*. *virens* to develop false smut balls? It is unlikely that local nutrients from stamens provide sufficient nutrients for *U*. *virens* to form false smut balls. As stamen filaments were infected and ultimately replaced by *U*. *virens* hyphae, and stamen filaments are connected to the base of ovary, and tap into rachilla via vascular bundles that transport nutrients from source organs (Wang *et al*., 1994), we speculated that *U*. *virens* hyphae may acquire abundant nutrients by establishing an interface at rachilla through stamen filaments. In turn, lacking of rice stamens could lead to the failure of establishing the proposed nutrient interface, preventing nutrient transport and blocking the formation of false smut balls.

To understand how *U*. *virens* manipulates rice stamens for successful infection, we further investigated *U*. *virens* modulation of nutrient‐related pathways in rice stamens via transcriptome analysis. Our data demonstrated that *U*. *virens* appears to manipulate rice stamens for nutrient acquisition in the following ways. First, *U*. *virens* upregulated the expression of sugar transporter genes, such as *SWEET11*, *SWEET14* and *SUT5* (Fig. S1; Table S1). SWEET11 and SWEET14 mediate efflux of glucose and can be hijacked by the bacterial phytopathogen *Xanthomonas oryzae* to pump sugars from host cells into apoplast space, which the pathogen inhabits (Chen *et al*., 2010). SUT5 has been demonstrated to transport sucrose (Sun *et al*., 2010). Upregulation of these genes in *U*. *virens*‐infected rice stamens likely facilitates sugar supply to support the intercellular growth of *U*. *virens*. Second, *U*. *virens* infection induced the expression of genes encoding galactinol synthase 2 and putative raffinose synthase, which are involved in biosynthesis of galactinol and raffinose family sugars (Smith *et al*., 1991) respectively (Fig. 6; Table S5). Raffinose could either be utilized directly by *U*. *virens* as a carbon source (Wang *et al*., 2016b) or in concert with galactinol, to biosynthesize stachyose. Stachyose has been shown to be a preferential carbon source for the growth of *U*. *virens* (Wang *et al*., 2016b), although rice genes responsible for stachyose biosynthesis have not been identified. Therefore, raffinose family sugars may be favourably manipulated by *U*. *virens*. Third, *U*. *virens* infection led to the upregulation of a subset of genes related to trehalose biosynthesis and hormone metabolisms of cytokinin and ethylene (Fig. 5; Table S4, Table S6). Trehalose could be utilized as a carbon source by *U*. *virens* (Fig. S3); additionally, trehalose may promote acquisition of nitrogen‐containing nutrients in *U*. *virens*‐inhabited intercellular space, as has been reported for *Pseudomonas aeruginosa* in plant leaf (Djonovic *et al*., 2013). Interestingly, increasing concentrations of cytokinin and ethylene, promoted by the rice endophyte *Phomopsis liquidambaris*, can also enhance the uptake of nitrogen‐containing nutrients in rice (Li *et al*., 2017). Thus, *U*. *virens* may stimulate rice to take up and supply with more nitrogen‐containing nutrients to appropriate locations to achieve pathogen proliferation, via modulating trehalose and hormone metabolisms. Finally, *U*. *virens* upregulated the expression of a subset of genes involved in the biosynthesis of amino acids (Fig. 6; Table S4), which may contribute to meeting the nitrogen requirements of *U*. *virens* growth.

Compared with previous transcriptome analyses on compatible interactions between different *U*. *virens* isolates and rice accessions, expressions of *SWEET11* and/or *SWEET14* were consistently upregulated by *U*. *virens* infection (Table S1)(Chao *et al*., 2014; Fan *et al*., 2015), suggesting that *SWEET* gene family in rice may also be targeted by the flower‐infecting fungal pathogen *U*. *virens*, in addition to the leaf‐infecting bacterial pathogen *Xanthomonas oryzae* pv. *oryzae* (Xu *et al*., 2019). Pathways associated with biotic stress, secondary metabolism and hormone metabolism were also consistently modulated in rice flowers infected with *U*. *virens*, according to the previous transcriptomic analyses and this study (Table S1) (Chao *et al*., 2014; Yang *et al*., 2014; Fan *et al*., 2015; Han *et al*., 2015). In a previous transcriptomic analysis (Fan *et al*., 2015), up to 35 rice grain‐filling‐related genes were activated by *U*. *virens* infection; whereas, in the present work, only three genes related to grain filling (*Os07g0182000*, *Os03g0188500* and *Os01g0644600*) were induced in rice stamens infected with *U*. *virens* (Table S1). Moreover, the present study uniquely identified, in rice stamen infected with *U*. *virens*, a subset of upregulated genes that are involved in the biosynthesis of trehalose and raffinose family sugars (Table S5), which may point to a novel nutrient acquisition strategy deployed by *U*. *virens*. These differences in expression patterns between different studies are probably attributable to the use of different rice accessions and/or different time points after *U*. *virens* inoculation for transcriptome analysis in these studies.

Overall, we demonstrated that *U*. *virens* requires rice stamens for successful colonization and development of false smut ball, and identified a set of candidate genes in rice stamens, which may be employed by *U*. *virens* to facilitate nutrient acquisition from the host. In the future, these candidate genes need to be further investigated to determine whether they are required for initial infection and/or false smut ball formation of *U*. *virens*.

## Experimental procedures

### *Plant materials and fungal isolates*

Rice accessions Pu6, W2555, X3107, Yixiang1B, and the corresponding floral mutants were grown in an experimental field of Sichuan Agricultural University, China. *Ustilaginoidea virens* isolate PJ52‐2‐5 was obtained from a rice false smut ball in Pu6 by amerosporous purification (Fan *et al*., 2019). The GFP‐tagged *U*. *virens* P4 was a gift from Prof. Yongfeng Liu from Jiangsu Academy of Agricultural Sciences, China.

### *Artificial inoculation*

Artificial inoculation of *U*. *virens* was conducted as described in a previous report (Fan *et al*., 2015). Briefly, *U*. *virens* mycelium was cultured on potato‐sucrose‐agar medium and transferred into potato‐sucrose broth (PSB) incubated at 28°C and 120 rpm for seven days. The blended mixture of mycelia and conidia (concentration adjusted to 1 × 10^6^ conidia/ml) was injected into rice panicles at late booting stages (5–7 days before heading) until the inoculum dripped out. PSB alone was inoculated into a separate set of panicles as mock‐inoculation controls.

### *Fluorescence microscopy and ESEM*

For fluorescence microscopy, rice spikelets were collected from P4‐inoculated panicles of Pu6 at multiple time points. Half of the lemma and palea was removed from spikelets for better observation of the inner floral organs under a fluorescence microscope (Zeiss Axio Imager A2, Carl Zeiss). For ESEM, spikelets were sampled from pistil‐ and/or stamen‐deficient floral mutants and their corresponding wild‐type plants, and directly examined with an environmental scanning electron microscope (FEI Quanta 450, FEI) at low‐vacuum (70 Pa), after removing the lemma and the palea.

### *Light microscopy and TEM*

Sample preparation for anatomical analysis was performed as previously described with minor modifications (Tang *et al*., 2013). In brief, *U*. *virens*‐infected spikelets were collected from *superwoman* mutant derived from EMS mutagenesis of Yixiang1B, and pre‐fixed in 2.5% glutaraldehyde in 0.1 M PBS (pH 7.0) overnight at 4°C. The pre‐fixed samples were washed three times with 0.1 M PBS (pH 7.0), 15–20 min each time, and post‐fixed with 1% osmium tetroxide. After a series of washing and dehydration steps, the samples were embedded in Spurr resin and polymerized overnight at 70°C.

For light microscopy, a series of 1 μm sections were prepared from the embedded samples, stained with 0.05% methylene blue, and observed under a Nikon Eclipse 80i microscope. For TEM, 6–7 nm ultrathin sections were prepared and examined under a TEM (Hitachi H‐7650) as previously described (Tang *et al*., 2013).

### *RNA‐Seq analysis*

Total RNAs were isolated from Pu6 rice stamens at 9 day post‐inoculation (dpi) of either P4 or PSB. Quantity and integrity of RNAs were analysed by using a Nanodrop 2000 spectrophotometer (Thermo Scientific, Waltham) and Bioanalyzer 2100 (Agilent Technologies, Santa Clara). Library construction and Illumina HiSeq 2500 sequencing were carried out at Novogene (Beijing, China). Sequencing data were filtered to remove adaptor and low‐quality reads. The generated clean data were then mapped to Oryza_sativa.IRGSP‐1.0.26 genome (Ensembl) by using TopHat2 with default parameters (Kim *et al*., 2013). Subsequently, gene expression was determined by HTSeq (Anders *et al*., 2015). Differential gene expression analysis was conducted with DESeq2 (Love *et al*., 2014), under the criteria of the absolute Log2 Fold change ≥1 and *P* < 0.05.

### *Quantitative PCR analysis*

Total RNAs were reverse transcribed with Primescript RT reagent kit with gDNA Eraser (Takara) according to the manufacturer's instructions and then subjected to quantitative PCR (qPCR) using SYBR Green Mix (Takara) and gene‐specific primers (Table S7). Rice gene *GAPDH* was used as the reference to calculate the relative expression of investigated genes using comparative C_T_ method 2^–ΔΔCT^ (Livak and Schmittgen, 2001).

## Supporting information


**Fig. S1.** Expression changes of sucrose‐starch and raffinose metabolism genes in rice stamens upon *Ustilaginoidea virens* infection. Differentially expressed genes (DEGs) (absolute Log2 Fold change≥1 and *P* value<0.05) were mapped to Sucrose‐starch metabolism (A) and Raffinose metabolism (B) pathways with MapMan software. Red points represent up‐regulated genes, while blue points represent down‐regulated ones. Expression data are included in Table S5.

**Fig. S2.** QPCR validation of differentially expressed genes randomly selected from RNA‐Seq analysis. Samples of rice stamens were collected from mock‐inoculated (CK) and *U*. *virens* P4‐infected (P4) spikelets, and subjected to qPCR analysis using rice *GAPDH* as the reference gene (Fan *et al*., 2015). Log2 Fold change of gene expression was presented. ‘‐Inf.’ indicates that expression Os06g0229800 was detected in CK, but not in P4‐infected stamens.

**Fig. S3.** Trehalose can be utilized by *Ustilaginoidea virens*. *U*. *virens* PJ52‐2‐5 was cultured at 28°C for 30 d in Czapek‐agar medium supplemented with a single carbon source of sucrose, or trehalose. Bar size = 1.5 cm.

**Table S1.** Differentially expressed genes in rice stamens upon *Ustilaginoidea virens* infection (absolute Log2 Fold change≥1 and *P* value<0.05).
**Table S2.** Pathway enrichment analysis of differentially expressed genes in rice stamens upon *Ustilaginoidea virens* infection.
**Table S3**. Differentially expressed genes involved in biotic stress or encoding receptor kinases.
**Table S4.** Differentially expressed genes involved in metabolism pathways.
**Table S5.** Differentially expressed genes involved in sucrose‐starch and raffinose metabolisms.
**Table S6**. Differentially expressed genes involved in hormone‐related pathways.
**Table S7**. Primers used in this study.

## References

[emi14881-bib-0001] Anders, S. , Pyl, P.T. , and Huber, W. (2015) HTSeq‐a Python framework to work with high‐throughput sequencing data. Bioinformatics 31: 166–169. 25260700 10.1093/bioinformatics/btu638 PMC4287950

[emi14881-bib-0002] Ashizawa, T. , Takahashi, M. , Arai, M. , and Arie, T. (2012) Rice false smut pathogen, *Ustilaginoidea virens*, invades through small gap at the apex of a rice spikelet before heading. J Gen Plant Pathol 78: 255–259.

[emi14881-bib-0003] Brooks, S.A. , Anders, M.M. , and Yeater, K.M. (2009) Effect of cultural management practices on the severity of false smut and kernel smut of rice. Plant Dis 93: 1202–1208. 30754580 10.1094/PDIS-93-11-1202

[emi14881-bib-0004] Chao, J. , Jin, J. , Wang, D. , Han, R. , Zhu, R. , Zhu, Y. , and Li, S. (2014) Cytological and transcriptional dynamics analysis of host plant revealed stage‐specific biological processes related to compatible rice‐*Ustilaginoidea virens* interaction. PLoS One 9: e91391.24646527

[emi14881-bib-0005] Chen, L.Q. , Hou, B.H. , Lalonde, S. , Takanaga, H. , Hartung, M.L. , Qu, X.Q. , *et al* (2010) Sugar transporters for intercellular exchange and nutrition of pathogens. Nature 468: 527–532. 21107422 10.1038/nature09606 PMC3000469

[emi14881-bib-0006] Djonovic, S. , Urbach, J.M. , Drenkard, E. , Bush, J. , Feinbaum, R. , Ausubel, J.L. , *et al* (2013) Trehalose biosynthesis promotes *Pseudomonas aeruginosa* pathogenicity in plants. PLoS Pathog 9: e1003217. 23505373 10.1371/journal.ppat.1003217 PMC3591346

[emi14881-bib-0007] Fan, J. , Du, N. , Li, L. , Li, G. , Wang, Y. , Zhou, Y. , *et al* (2019) A core effector UV_1261 promotes *Ustilaginoidea virens* infection via spatiotemporally suppressing plant defense. Phytopathol Res 1: 11.

[emi14881-bib-0008] Fan, J. , Guo, X.Y. , Huang, F. , Li, Y. , Liu, Y.‐F. , Li, L. , *et al* (2014) Epiphytic colonization of *Ustilaginoidea virens* on biotic and abiotic surfaces implies the widespread presence of primary inoculum for rice false smut disease. Plant Pathol 63: 937–945.

[emi14881-bib-0009] Fan, J. , Guo, X.Y. , Li, L. , Huang, F. , Sun, W.X. , Li, Y. , *et al* (2015) Infection of *Ustilaginoidea virens* intercepts rice seed formation but activates grain‐filling‐related genes. J Integr Plant Biol 57: 577–590. 25319482 10.1111/jipb.12299 PMC5024071

[emi14881-bib-0010] Fan, J. , Yang, J. , Wang, Y.Q. , Li, G.B. , Li, Y. , Huang, F. , and Wang, W.‐M. (2016a) Current understanding on *Villosiclava virens*, a unique flower‐infecting fungus causing rice false smut disease. Mol Plant Pathol 17: 1321–1330. 26720072 10.1111/mpp.12362 PMC6638446

[emi14881-bib-0011] Fan, L.L. , Yong, M.L. , Li, D.Y. , Liu, Y.J. , Lai, C.H. , Chen, H.M. , *et al* (2016b) Effect of temperature on the development of sclerotia in *Villosiclava virens* . J Integr Agric 15: 2550–2555.

[emi14881-bib-0012] Fan, R. , Wang, Y. , Liu, B. , Zhang, J. , Wang, H. , and Hu, D. (2010) The process of asexual spore formation and examination of chlamydospore germination of *Ustilaginoidea virens* . Mycosystema 29: 188–192.

[emi14881-bib-0013] Han, Y. , Zhang, K. , Yang, J. , Zhang, N. , Fan, A. , Zhang, Y. , *et al* (2015) Differential expression profiling of the early response to *Ustilaginoidea virens* between false smut resistant and susceptible rice varieties. BMC Genomics 16: 955. 26573512 10.1186/s12864-015-2193-x PMC4647755

[emi14881-bib-0014] Hu, M. , Luo, L. , Wang, S. , Liu, Y. , and Li, J. (2014) Infection processes of *Ustilaginoidea virens* during artificial inoculation of rice panicles. Eur J Plant Pathol 139: 67–77.

[emi14881-bib-0015] Ikegami, H. (1960) Studies on the false smut of Rice, IV. Infection of the false smut due to inoculation with chlamydospores and ascospores at the booting stage of rice plants. Res Bull Fac Agric Gifu Univ 12: 45–51.

[emi14881-bib-0016] Ikegami, H. (1963) Studies on the false smut of rice X. invasion of chlamydospores and hyphae of the false smut fungus into rice plants. Res Bull Fac Agric Gifu Univ 18: 54–60.

[emi14881-bib-0017] Jecmen, A.C. , and TeBeest, D.O. (2015) First report of the occurrence of a white smut infecting rice in Arkansas. J Phytopathol 163: 138–143.

[emi14881-bib-0018] Kim, D. , Pertea, G. , Trapnell, C. , Pimentel, H. , Kelley, R. , and Salzberg, S.L. (2013) TopHat2: accurate alignment of transcriptomes in the presence of insertions, deletions and gene fusions. Genome Biol 14: R36. 23618408 10.1186/gb-2013-14-4-r36 PMC4053844

[emi14881-bib-0019] Koiso, Y. , Li, Y. , Iwasaki, S. , Hanaoka, K. , Kobayashi, T. , Sonoda, R. , *et al* (1994) Ustiloxins, antimitotic cyclic peptides from false smut balls on rice panicles caused by *Ustilaginoidea virens* . J Antibiot 47: 765–773. 8071121 10.7164/antibiotics.47.765

[emi14881-bib-0020] Ladhalakshmi, D. , Laha, G.S. , Singh, R. , Karthikeyan, A. , Mangrauthia, S.K. , Sundaram, R.M. , *et al* (2012) Isolation and characterization of *Ustilaginoidea virens* and survey of false smut disease of rice in India. Phytoparasitica 40: 171–176.

[emi14881-bib-0021] Li, S.C. , Li, W.B. , Huang, B. , Cao, X.M. , Zhou, X.Y. , Ye, S.M. , *et al* (2013a) Natural variation in PTB1 regulates rice seed setting rate by controlling pollen tube growth. Nat Commun 4: 2793. 24240868 10.1038/ncomms3793

[emi14881-bib-0022] Li, W. , Li, L. , Feng, A. , Zhu, X. , and Li, J. (2013b) Rice false smut fungus, *Ustilaginoidea virens*, inhibits pollen germination and degrades the integuments of rice ovule. Am J Plant Sci 4: 2295–2304.

[emi14881-bib-0023] Li, X. , Zhou, J. , Xu, R.‐S. , Meng, M.‐Y. , Yu, X. , and Dai, C.‐C. (2017) Auxin, cytokinin, and ethylene involved in rice N availability improvement caused by endophyte *Phomopsis liquidambaris* . J Plant Growth Regul 37: 128–143.

[emi14881-bib-0024] Liao, Y.X. , Bai, Q. , Xu, P.Z. , Wu, T.K. , Guo, D.M. , Peng, Y.B. , *et al* (2018) Mutation in rice Abscisic Acid2 results in cell death, enhanced disease‐resistance, altered seed dormancy and development. Front Plant Sci 9: 405. 29643863 10.3389/fpls.2018.00405 PMC5882781

[emi14881-bib-0025] Livak, K.J. , and Schmittgen, T.D. (2001) Analysis of relative gene expression data using real‐time quantitative PCR and the 2(T)(−delta delta C) method. Methods 25: 402–408. 11846609 10.1006/meth.2001.1262

[emi14881-bib-0026] Love, M.I. , Huber, W. , and Anders, S. (2014) Moderated estimation of fold change and dispersion for RNA‐seq data with DESeq2. Genome Biol 15: 550. 25516281 10.1186/s13059-014-0550-8 PMC4302049

[emi14881-bib-0027] Lu, S. , Sun, W. , Meng, J. , Wang, A. , Wang, X. , Tian, J. , *et al* (2015) Bioactive bis‐naphtho‐gamma‐pyrones from rice false smut pathogen *Ustilaginoidea virens* . J Agric Food Chem 63: 3501–3508. 25781489 10.1021/acs.jafc.5b00694

[emi14881-bib-0028] Nagasawa, N. , Miyoshi, M. , Sano, Y. , Satoh, H. , Hirano, H. , Sakai, H. , and Nagato, Y. (2003) *SUPERWOMAN1* and *DROOPING LEAF* genes control floral organ identity in rice. Development 130: 705–718. 12506001 10.1242/dev.00294

[emi14881-bib-0029] Nakamura, K. , Izumiyama, N. , Ohtsubo, K. , Koiso, Y. , Iwasaki, S. , Sonoda, R. , *et al* (1994) "Lupinosis"‐like lesions in mice caused by ustiloxin, produced by *Ustilaginoidea virens*: a morphological study. Nat Toxins 2: 22–28. 8032691 10.1002/nt.2620020106

[emi14881-bib-0030] Prakobsub, K. , and Ashizawa, T. (2017) Intercellular invasion of rice roots at the seedling stage by the rice false smut pathogen, *Villosiclava virens* . J Gen Plant Pathol 83: 358–361.

[emi14881-bib-0031] Rong, N. , Yong, M. , Ying, X.U. , and Hu, D.W. (2017) Relationship between ultrastructure of cell walls of rice spikelets and infection specificity of *Villosiclava virens* . Acta Bot Boreali‐Occident Sin 37: 1–7.

[emi14881-bib-0032] Smith, P.T. , Kuo, T.M. , and Crawford, C.G. (1991) Purification and characterization of galactinol synthase from mature zucchini squash leaves. Plant Physiol 96: 693–698. 16668244 10.1104/pp.96.3.693 PMC1080832

[emi14881-bib-0033] Song, J.H. , Wei, W. , Lv, B. , Lin, Y. , Yin, W.X. , Peng, Y.L. , *et al* (2016) Rice false smut fungus hijacks the rice nutrients supply by blocking and mimicking the fertilization of rice ovary. Environ Microbiol 18: 3840–3849. 27129414 10.1111/1462-2920.13343

[emi14881-bib-0034] Sun, W.B. , Wang, A. , Xu, D. , Wang, W.X. , Meng, J.J. , Dai, J.G. , *et al* (2017) New ustilaginoidins from rice false smut balls caused by *Villosiclava virens* and their phytotoxic and cytotoxic activities. J Agric Food Chem 65: 5151–5160. 28574707 10.1021/acs.jafc.7b01791

[emi14881-bib-0035] Sun, Y. , Reinders, A. , LaFleur, K.R. , Mori, T. , and Ward, J.M. (2010) Transport activity of rice sucrose transporters OsSUT1 and OsSUT5. Plant Cell Physiol 51: 114–122. 19965875 10.1093/pcp/pcp172 PMC2807175

[emi14881-bib-0036] Tang, Y.X. , Jin, J. , Hu, D.W. , Yong, M.L. , Xu, Y. , and He, L.P. (2013) Elucidation of the infection process of *Ustilaginoidea virens* (teleomorph: *Villosiclava virens*) in rice spikelets. Plant Pathol 62: 1–8.

[emi14881-bib-0037] Wang, W.M. , Fan, J. , and Jeyakumar, J.M.J. (2019) Rice false smut: an increasing threat to grain yield and quality In Protecting Rice Grains in the Post‐Genomic Era, Jia Y. (ed). London, UK: IntechOpen. pp. 89–108.

[emi14881-bib-0038] Wang, X. , Fu, X. , Lin, F. , Sun, W. , Meng, J. , Wang, A. , *et al* (2016a) The contents of ustiloxins A and B along with their distribution in rice false smut balls. Toxins 8: 262. 10.3390/toxins8090262 PMC5037488 27608042

[emi14881-bib-0039] Wang, X.H. , Wang, J. , Lai, D.W. , Wang, W.X. , Dai, J.G. , Zhou, L.G. , and Liu, Y. (2017) Ustiloxin G, a new cyclopeptide mycotoxin from rice false smut balls. Toxins 9. 10.3390/toxins9020054 PMC5331433 28208606

[emi14881-bib-0040] Wang, Y.Q. , Li, G.B. , Gong, Z.Y. , Li, Y. , Huang, F. , Fan, J. , and Wang, W.M. (2016b) Stachyose is a preferential carbon source utilized by the rice false smut pathogen, *Villosiclava virens* . Physiol Mol Plant Pathol 96: 69–76.

[emi14881-bib-0041] Wang, Z. , Gu, Y. , and Gao, Y. (1994) Structure of rachi lae and its changes during flower opening and closure in rice. J Jiangsu Agric Coll 15: 1–10.

[emi14881-bib-0042] Xu, Z. , Xu, X. , Gong, Q. , Li, Z. , Li, Y. , Wang, S. , *et al* (2019) Engineering broad‐spectrum bacterial blight resistance by simultaneously disrupting variable TALE‐binding elements of multiple susceptibility genes in rice. Mol Plant 12: 1434–1446. 31493565 10.1016/j.molp.2019.08.006

[emi14881-bib-0043] Yang, C. , Li, L. , Feng, A. , Zhu, X. , and Li, J. (2014) Transcriptional profiling of the responses to infection by the false smut fungus *Ustilaginoidea virens* in resistant and susceptible rice varieties. Can J Plant Pathol 36: 377–388.

[emi14881-bib-0044] Yong, M.L. , Deng, Q.D. , Fan, L.L. , Miao, J.K. , Lai, C.H. , Chen, H.M. , *et al* (2018) The role of *Ustilaginoidea virens* sclerotia in increasing incidence of rice false smut disease in the subtropical zone in China. Eur J Plant Pathol 150: 669–677.

[emi14881-bib-0045] Yong, M.L. , Fan, L.L. , Li, D.Y. , Liu, Y.J. , Cheng, F.M. , Xu, Y. , *et al* (2016) *Villosiclava virens* infects specifically rice and barley stamen filaments due to the unique host cell walls. Microsc Res Tech 79: 838–844. 27357263 10.1002/jemt.22710

[emi14881-bib-0046] Zhou, L. , Lu, S. , Shan, T. , Wang, P. , Sun, W. , Chen, Z. , and Wang, S. (2012) Chemistry and biology of mycotoxins from rice false smut pathogen In Mycotoxins: Properties, Applications and Hazards, Melborn B.J., and Greene J.C. (eds). New York, NY: Nova Science Publishers, pp. 109–130.

