# Supplementary figures and images for: The false smut pathogen Ustilaginoidea virens requires rice stamens for false smut ball formation
Source: Environ Microbiol. 2019 Dec 11;22(2):646–59. doi: 10.1111/1462-2920.14881 (PMC7028044; doi:10.1111/1462-2920.14881)

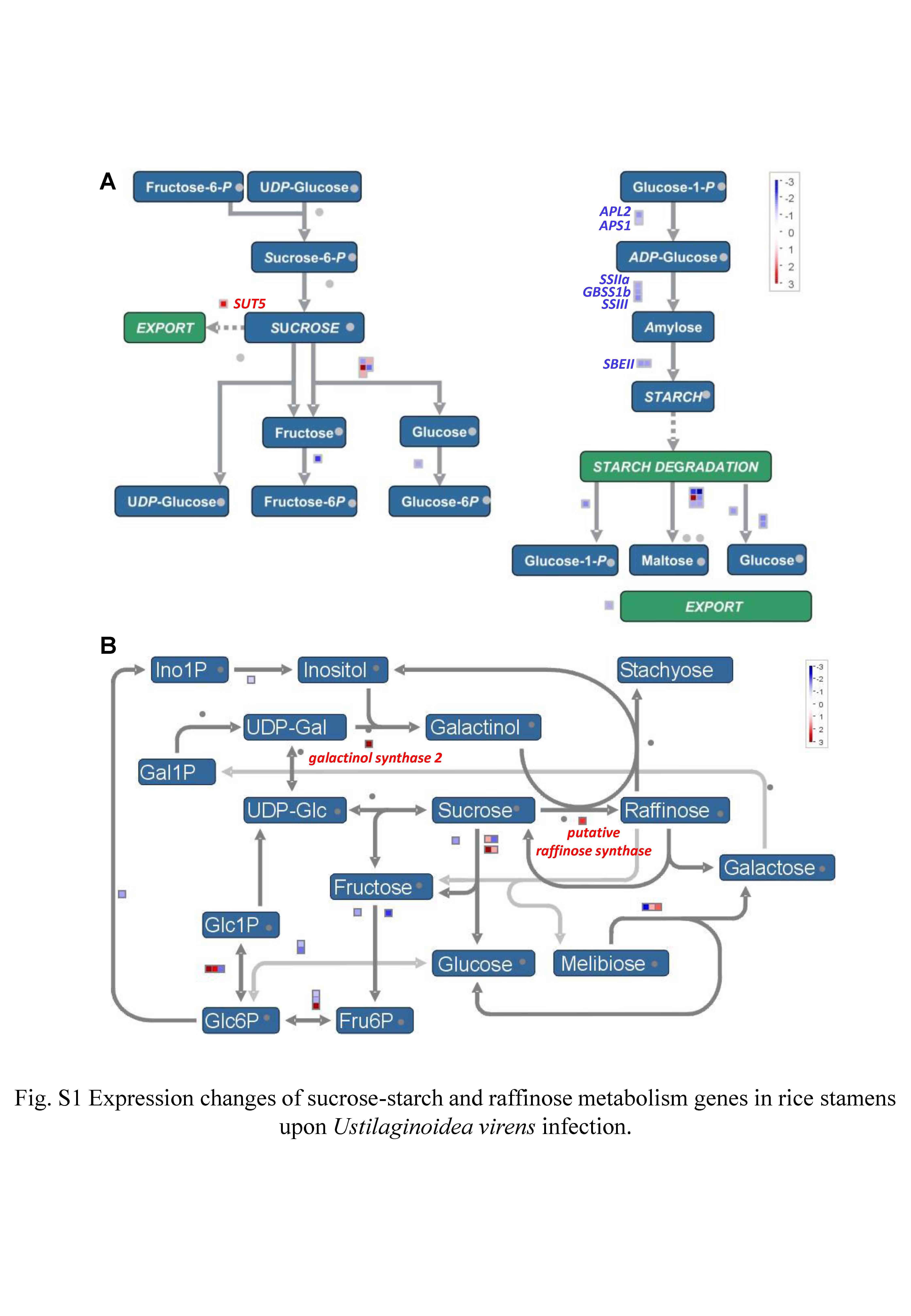

Supplement: Supplementary file 1 — Fig. S1. Expression changes of sucrose‐starch and raffinose metabolism genes in rice stamens upon Ustilaginoidea virens infection. Differentially expressed genes (DEGs) (absolute Log2 Fold change≥1 and P value<0.05) were mapped to Sucrose‐starch metabolism (A) and Raffinose metabolism (B) pathways with MapMan software. Red points represent up‐regulated genes, while blue points represent down‐regulated ones. Expression data are included in Table S5. [file EMI-22-646-s001.tif]

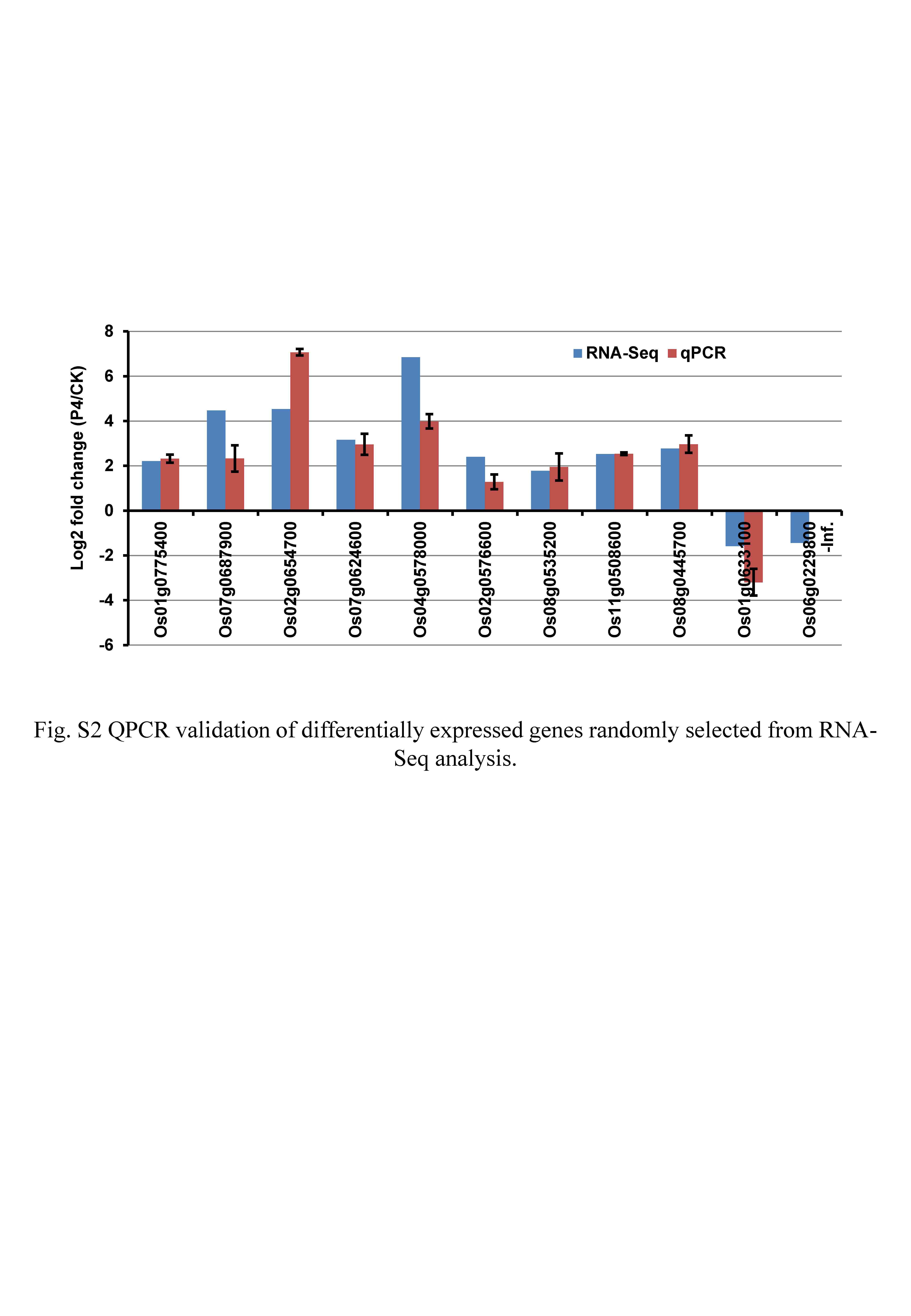

Supplement: Supplementary file 2 — Fig. S2. QPCR validation of differentially expressed genes randomly selected from RNA‐Seq analysis. Samples of rice stamens were collected from mock‐inoculated (CK) and U. virens P4‐infected (P4) spikelets, and subjected to qPCR analysis using rice GAPDH as the reference gene (Fan et al., 2015). Log2 Fold change of gene expression was presented. ‘‐Inf.’ indicates that expression Os06g0229800 was detected in CK, but not in P4‐infected stamens. [file EMI-22-646-s002.tif]

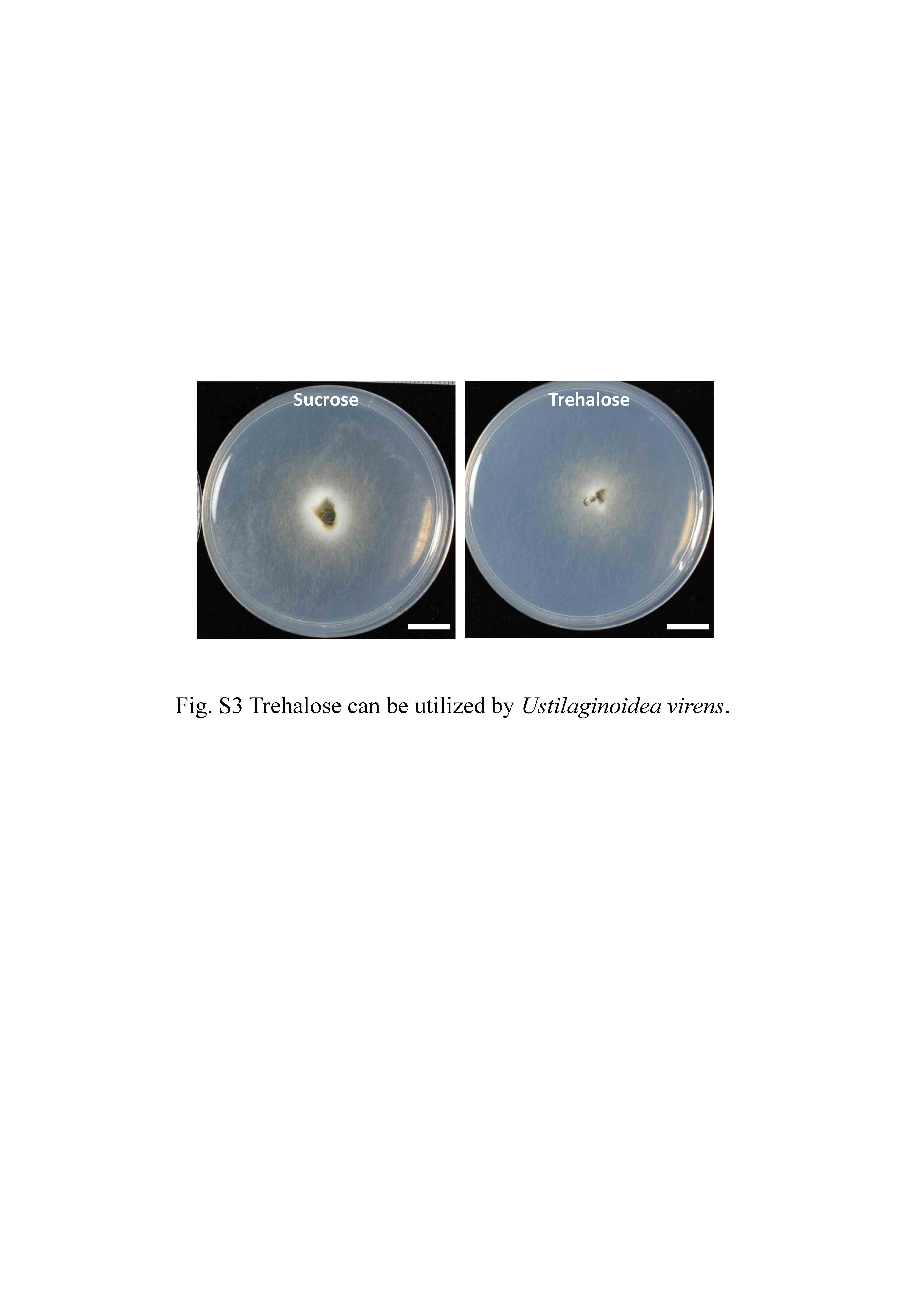

Supplement: Supplementary file 3 — Fig. S3. Trehalose can be utilized by Ustilaginoidea virens. U. virens PJ52‐2‐5 was cultured at 28°C for 30 d in Czapek‐agar medium supplemented with a single carbon source of sucrose, or trehalose. Bar size = 1.5 cm. [file EMI-22-646-s003.tif]
